# Curculigoside Attenuates Endoplasmic Reticulum Stress-Induced Epithelial Cell and Fibroblast Senescence by Regulating the SIRT1-P300 Signaling Pathway

**DOI:** 10.3390/antiox13040420

**Published:** 2024-03-29

**Authors:** Weixi Xie, Lang Deng, Rui Qian, Xiaoting Huang, Wei Liu, Siyuan Tang

**Affiliations:** 1 Xiangya Nursing School, Central South University, Changsha 410013, China; 18508450233@163.com (W.X.); dengl036@163.com (L.D.); qianrui521958@outlook.com (R.Q.); xiaotinghuang@csu.edu.cn (X.H.); 2 The School of Nursing, Ningxia Medical University, Yinchuan 750004, China

**Keywords:** curculigoside, GSK3β, senescence, pulmonary fibrosis, endoplasmic reticulum stress

## Abstract

The senescence of alveolar epithelial cells (AECs) and fibroblasts plays a pivotal role in the pathogenesis of idiopathic pulmonary fibrosis (IPF), a condition lacking specific therapeutic interventions. Curculigoside (CCG), a prominent bioactive constituent of *Curculigo*, exhibits anti-osteoporotic and antioxidant activities. Our investigation aimed to elucidate the anti-senescence and anti-fibrotic effects of CCG in experimental pulmonary fibrosis and delineate its underlying molecular mechanisms. Our findings demonstrate that CCG attenuates bleomycin-induced pulmonary fibrosis and lung senescence in murine models, concomitantly ameliorating lung function impairment. Immunofluorescence staining for senescence marker p21, alongside SPC or α-SMA, suggested that CCG’s mitigation of lung senescence correlates closely with the deceleration of senescence in AECs and fibroblasts. In vitro, CCG mitigated H_2_O_2_-induced senescence in AECs and the natural senescence of primary mouse fibroblasts. Mechanistically, CCG can upregulate SIRT1 expression, downregulating P300 expression, enhancing Trim72 expression to facilitate P300 ubiquitination and degradation, reducing the acetylation levels of antioxidant enzymes, and upregulating their expression levels. These actions collectively inhibited endoplasmic reticulum stress (ERS) and alleviated senescence. Furthermore, the anti-senescence effects and mechanisms of CCG were validated in a D-galactose (D-gal)-induced progeroid model. This study provides novel insights into the mechanisms underlying the action of CCG in cellular senescence and chronic diseases, offering potential avenues for the development of innovative drugs or therapeutic strategies.

## 1. Introduction

Idiopathic pulmonary fibrosis (IPF) is a severe and progressive lung disease characterized by increased myofibroblast activity and the abnormal accumulation of extracellular matrix (ECM) in lung tissues [1]. It is closely associated with various age-related processes and cellular dysregulations that lead to maladaptation to stress and susceptibility to lung fibrosis [2]. There is growing evidence suggesting that cellular senescence drives the occurrence and progression of lung fibrosis [3,4]. Cellular senescence is defined as the permanent arrest of the cell cycle and functional disruption [5]. Senescent cells secrete a plethora of active substances, collectively known as the senescence-associated secretory phenotype (SASP), which influences the cellular microenvironment. In the context of pulmonary fibrosis, the SASP secretes inflammatory factors, chemokines, growth factors, and matrix metalloproteinases, further contributing to lung tissue damage and fibrosis [6,7]. Currently FDA-approved drugs for treating IPF, such as pirfenidone and nintedanib, have limited efficacy and safety concerns [8]. Therefore, there is an urgent need to develop new therapeutic approaches that are both effective and safe based on the cellular senescence characteristics in lung fibrosis.

Alveolar epithelial cells (AECs) and fibroblasts, as primary targets of senescence in pulmonary fibrosis, maintain lung function stability [9,10]. Single-cell RNA sequencing studies have revealed enhanced senescence of AEC2 cells in the lung tissue of IPF patients, activating pro-fibrotic myofibroblasts through multiple typical pathways, thereby accelerating the progression of pulmonary fibrosis [11]. In mouse models, reducing or reversing the senescence of AECs effectively alleviates bleomycin (BLM)-induced pulmonary fibrosis in mice [12]. Primary fibroblasts isolated from the lungs of IPF patients exhibit a more pronounced senescence phenotype [13]. Additionally, clearing senescent lung fibroblasts can alleviate BLM-induced pulmonary fibrosis in mice [14]. Therefore, mitigating the senescence of AECs and fibroblasts is a key strategy for the treatment of pulmonary fibrosis.

The activation of endoplasmic reticulum stress (ERS) and unfolded protein response (UPR) is closely associated with age-related lung diseases. Impaired endoplasmic reticulum (ER) function leads to the accumulation of misfolded proteins, triggering the activation of the unfolded protein response (UPR) [15]. Initially, UPR activation serves to protect cells. The increased expressions of proteins such as ATF4, CHOP, and BIP, which are associated with ERS, reflect that cells are under stress and are coping with impaired ER function or increased burden [16]. However, if ERS is excessive or prolonged, it can lead to cellular senescence [17]. Substantial evidence indicates that ERS is prevalent in the lungs of patients with IPF, predominantly occurring in AECs and fibroblasts [18,19]. Consistently, in a BLM-induced mouse model of lung fibrosis, the inhibition of ERS effectively reduces cellular senescence [20]. ERS is closely associated with oxidative stress. Excessive reactive oxygen species (ROS) in senescent cells disrupt the redox balance of the ER, leading to ERS/UPR [21]. Moreover, ERS/UPR exacerbates ROS generation, forming a vicious cycle that intensifies cellular senescence [22].

Antioxidant enzymes play a crucial role in maintaining redox balance by scavenging ROS, thereby protecting organs from damage and fibrosis [23]. However, severe lung stimuli such as BLM can impair the antioxidant enzyme system, leading to ROS accumulation and accelerating lung fibrosis progression [24]. The expression and activity of antioxidant enzymes are regulated by various pathways, with a crucial balance between acetylation and deacetylation [25,26]. SIRT1 (Sirtuin 1) is a NAD+-dependent protein deacetylase that plays a crucial role in regulating the cellular lifespan, metabolism, and stress response [27]. P300, as a histone acetyltransferase, is essential for gene transcription, cell proliferation, and differentiation [28]. Extensive research suggests that the SIRT1-P300 signaling pathway regulates the acetylation balance of antioxidant enzymes within cells, making it a vital pathway for antioxidation and anti-aging, closely associated with fibrosis treatment [29,30,31]. Despite the acknowledged significance of the SIRT1-P300 signaling pathway in fibrosis, its functional mechanism remains unclear. Studies on small-molecule compounds targeting the SIRT1-P300 signaling pathway for the treatment of pulmonary fibrosis are scarce. Therefore, exploring and developing drug strategies targeting the SIRT1-P300 signaling pathway is crucial for combating fibrosis.

CCG is an active compound extracted from the traditional Chinese herb *Curculigo*. Reports suggest that CCG exhibits significant biological effects, including immunomodulation, antioxidative properties, and anti-osteoporosis effects, and herbal formulations primarily containing *Curculigo* exert anti-aging activity [32,33,34]. In preliminary experiments, we found that the SIRT1-P300 signaling pathway mediates the anti-aging effects of CCG. Therefore, this study aimed to investigate the impact and mechanisms of CCG on the SIRT1-P300 signaling pathway in vitro and in vivo.

## 2. Materials and Methods

### 2.1. Animals Experiments

C57BL/6 mice (male, 8 weeks) were obtained from the Department of Animal, Central South University, and grouped according to different purposes. All animal experimental protocols are approved by the Ethics Committee of Central South University (Certificate No. CSU-2022-0219; Changsha, China).

C57BL/6 J mice were randomly divided into a CON group, BLM group, BLM + 0.6 mg/kg group, BLM + 3 mg/kg group, and BLM + 15 mg/kg group. After one week of adaptive feeding, mice were tracheal-injected with 30 μL saline or 3 mg/kg bleomycin (Nippon Kayaku, Tokyo, Japan). Starting from day 14, mice were injected continuously intraperitoneally with different concentrations of CCG or an equivalent amount of saline. On day 28, the mice were anesthetized and subsequent experiments on lung fibrosis were performed.

A total of 30 mice were randomly divided into a CON group, D-gal group, D-gal + 0.6 mg/kg group, D-gal + 3 mg/kg group, and D-gal + 15 mg/kg group. Mice in the D-gal group and D-gal + 0.6, 3, and 15 mg/kg groups were subcutaneously injected with D-gal (150 mg/kg, dissolved in physiological saline, Sigma-Aldrich, Saint Louis, MO, USA) through the neck and back every day, and intraperitoneally injected with physiological saline or CCG daily. After six weeks, the mice were anesthetized through an intraperitoneal injection of pentobarbital sodium to perform subsequent experiments.

Curculigoside was bought from Selleck (Shanghai, China), its purity was more than 99.85% (Supplementary Figure S2).

### 2.2. Histological Analysis

Lung tissue was collected in lobes, and the right upper section was embedded with paraffin. And these lungs were prepared for hematoxylin–eosin (H&E) staining and Masson’s trichrome staining to detect pathological changes in the lung.

### 2.3. Ashcroft Scores

Six researchers of related fields were assembled to observe different groups of lung sections. The researchers scored the sections individually according to Ashcroft scoring rules, and these scores were collected and analyzed statistically.

### 2.4. Survival Rate

The survival of the mice was recorded from the 15th day after the mice were injected with bleomycin. Different groups of mice were checked and recorded daily, with surviving mice recorded as “1” and dead mice recorded as “0”. After 14 consecutive days of recording, the mice were sacrificed, and all data were statistically analyzed.

### 2.5. Measurement of Hydroxyproline Levels

A fraction of lung tissue was weighed and measured with a hydroxyproline assay kit (Jiancheng Bioengineering Institute, Nanjing, China) according to the instructions.

### 2.6. Respiratory Function

Mice were subjected to tracheal intubation under anesthesia. We measured the breathing frequency, tidal volume (TV), lung volume (LV), and minute volume (MV) of the mice using the BUXCO system (Max Ⅱ, Buxco Electronics, Inc., Wilmington, NC, USA).

### 2.7. Immunofluorescence

Lung tissue was sliced after paraformaldehyde fixation and dehydration embedding. After antigen repairing and the blocking of endogenous peroxidase, sections were blocked with 5% BSA (Thermo Fisher Scientific, Waltham, MA, USA). For cultured cells, they were also blocked after fixation and permeabilization with Triton X-100 (Sigma-Aldrich, USA). After that, sections of cells were incubated with primary antibody (α-SMA, Proteintech, Rosemont, IL, USA, 1:200; P21, Abcam, Cambridge, UK, 1:100; SPC, Abclonal, Woburn, MA, USA, 1:200; Pho-GSK3β, Proteintech, 1:100; P53, Proteintech, 1:100) at 4 °C overnight. Washed with PBS 3 times, they were incubated at room temperature with secondary antibody for 1 h, and the nuclei were stained with DAPI (Solarbio, Beijing, China). After sealing the sections, the sections were observed under a fluorescence microscope (Nikon, Tokyo, Japan).

### 2.8. β-Galactosidase Staining

Cells were washed once with PBS after different treatments. After 15 min of fixation, the cells were washed three times with PBS and stained with working solution and incubated overnight at 37 °C. The instructions of the Senescence-Associatedβ-Galactosidase (SA-β-Gal) Stain Kit (Solarbio Life Science, Beijing, China) were followed throughout, and the cells were observed under a light microscope (Nikon, Japan).

### 2.9. Western Blotting (WB)

Proteins were extracted from ground lung tissue or cultured cell samples by adding RIPA lysate (Solarbio Life Science, China). The protein concentration was determined with a PierceTM BCA Protein Assay Kit (Thermo Fisher Scientific, Waltham, MA, USA) after an addition of a phosphatase inhibitor and a protease inhibitor (APExBIO Technology LLC, Houston, TX, USA) according to the volume of the sample. Proteins were heated to 100 °C for denaturation and then electrophoresed in 10% SDS-PAGE gels. The proteins were transferred with a 0.2 μm PVDF membrane (Millipore, Burlington, MA, USA) and blocked with 5% skimmed milk (Sigma-Aldrich, USA) for 2 h. After elution, the samples were incubated with the corresponding primary antibody at 4 °C overnight. After 1 h of secondary antibody (Proteintech, Shanghai, China) incubation at room temperature, the proteins were visualized with ECL luminescent solution (Cwbio, Taizhou, China) in a GeneGnome XRQ imager (Syngene, Cambridge, UK). The gray values of the bands were calculated and analyzed using ImageJ 1.5.2a software. The primary antibodies used in this experiment are shown in Table 1.

### 2.10. Quantitative Real-Time PCR (qPCR)

Total RNA was extracted from tissues or cultured cells through Trizol using the TransZol Kit (TransGen Biotech, Beijing, China) and reverse transcribed using the NovoScript^®^Plus All-in-one 1st Strand cDNA Synthesis SuperMix (Novoprotein, Shanghai, China). The concentration and purity of the cDNA were determined using Varioskan LUX (Thermo Fisher, USA), and real-time quantitative PCR was performed on a Bio-Rad CFX96 Touch Real-Time PCR Derection System (Bio-Rad, Hercules, CA, USA) using the NovoStart^®^ Fast SYBR qPCR SuperMix (Novoprotein, China). Primers were obtained from Sangong Biotechnology Co., Shanghai, China, and the sequences are shown in Table 2.

### 2.11. siRNA Transfection

Sirt1 siRNA (m) (sc-20987), Trim72 siRNA (m) (sc-154670), and control siRNA (sc-36869) were bought from Santa Cruz (Santa Cruz, CA, USA). Cells were transfected using LipofectamineTM 3000 (Thermo Fisher, USA) to knockdown the genes of Sirt1 and Trim72. After treating the cells with drugs, they were analyzed using Western blotting, immunofluorescence, β-galactosidase staining, ROS staining, and fluo-3AM staining.

### 2.12. Immunoprecipitation (IP)

To assess the interactions between Ac-lysine and CAT, Ac-lysine and SOD1, and Ac-lysine and SOD2, the following steps were taken: cells were washed three times with PBS and lysed on ice for 1 h using a lysis buffer containing complete protease inhibitor PMSF (Solarbio, China). After collecting the cells, centrifugation was performed at 12,000 rpm for 10 min at 4 °C. The resulting supernatant was incubated with Dynabeads™ Protein G (Invitrogen, Carlsbad, CA, USA) for 3 h at 4 °C, followed by centrifugation. Dynabead separation was achieved using DynaMag™-2 (Invitrogen, USA). The supernatant was supplemented with Ac-lysine polyclonal antibody (Proteintech, China) and incubated overnight at 4 °C. Dynabeads were separated again using DynaMag™-2 and subjected to 3 washes with lysis buffer. Subsequently, immunoprecipitated proteins were analyzed via Western blotting.

### 2.13. ROS Level

The BALFs from mice or cultured cells were centrifuged, and the supernatant was removed to obtain cell precipitates. To detect the content of ROS, the precipitates were incubated with H2DCFCDA (Thermo Fisher, USA) for 30 min at room temperature in dark. The level of ROS was detected via fluorescence microscopy (Nikon, Japan) and flow cytometry (BD LSRFortessa, Pleasanton, CA, USA).

### 2.14. Detection of Calcium Influx

Treated cells were incubated with HBSS solution (Pricella, Wuhan, China) in Fluo-3AM working solution (Solarbio, China) for 20 min at 37 °C. After being washed with HEPES buffer saline (Pricella, China), the cells were incubated with HBSS containing 1% fetal bovine serum (Pricella, China) for 40 min. After being washed, the cells were photographed under the fluorescence microscope (Nikon, Japan) or resuspended with HEPES solution and detected via flow cytometry (BD LSRFortessa, USA).

### 2.15. Network Pharmacology and Molecular Docking

Target data for CCG were obtained from ITCM (http://itcm.biotcm.net/) (accessed on 15 December 2023), ETCM (http://www.tcmip.cn/ETCM/) (accessed on 15 December 2023), and TCMSP (https://tcmsp-e/com/) (accesed on 15 December 2023), and target data for IPF were obtained through OMIM (http://omim.org) (accessed on 15 December 2023) and DisGeNET (http://www.disgenet.org/) (accessed on 15 December 2023). The intersection of the two datasets was obtained through Venny 2.1 (https://bioinfogp.cnb.csic.es/tools/venny/index.html) (accessed on 5 December 2023), and scores were calculated using String Database (https://string-db.org/) (accessed on 15 December 2023) and Cytoscape 3.9.1.

### 2.16. Cellular Thermal Shift Assay (CESTA)

After resuspending AECs cells treated with or without 10 μM CCG in pre-chilled PBS, they were divided into 6 groups and subjected to heat treatment at different temperatures (40 °C, 43 °C, 46 °C, 49 °C, 52 °C, and 55 °C). Subsequently, cell lysis was performed using NP-40 (Solarbio, China), and the stability of GSK3β at different temperatures was assessed via Western blotting.

### 2.17. Cell Culture

Primary mouse lung fibroblasts were obtained using a digestion method. Mice were anesthetized with sodium pentobarbital, and their hearts were lavaged with pre-chilled PBS. Lung tissues were then excised, placed in pre-chilled PBS, and cut into 1–2 cm^2^ pieces. The lung tissue was digested in DMEM digestion medium containing 1 mg/mL collagenase I at 37 °C for 1 h. Following digestion, cells were passed through 70 and 40 μm cell filters, centrifuged, and resuspended in a DMEM high-glucose medium supplemented with 20% fetal bovine serum (FBS, Sigma-Aldrich) and 1% penicillin/streptomycin (Gibco, Grand Island, NY, USA). Cells were cultured at 37 °C in a humidified atmosphere with 5% CO_2_. The identification of primary fibroblasts was confirmed using Vimentin staining (Supplementary Figure S1).

MLE12 cells (ATCC, Manassas, VA, USA) were cultured at 37 °C in a humidified atmosphere with 5% CO_2_ using complete medium containing 10% FBS (Gibco) and 1% penicillin/streptomycin (Procell Life Science & Technology, Wuhan, China). MLE12 cells were seeded into a 12-well plate (1 × 10^5^ cells per well) and cultured for 24 h until the cell density reached 60%. The cells were then stimulated with H_2_O_2_ (100 μM, Sigma-Aldrich, USA) for 2 h, followed by washing twice with PBS, and further incubated with fresh medium (with or without CCG) for 72 h.

### 2.18. Statistical Analysis

All the data were represented as the mean ± SD and analyzed with GraphPad Prism 9.0 software. For data that conformed to a normal distribution, one-way ANOVA was used for comparisons between groups. It was considered statistically significant when the *p*-value was less than 0.05.

## 3. Results

### 3.1. CCG Alleviated BLM-Induced Pulmonary Fibrosis in Mice

**Figure 1:**
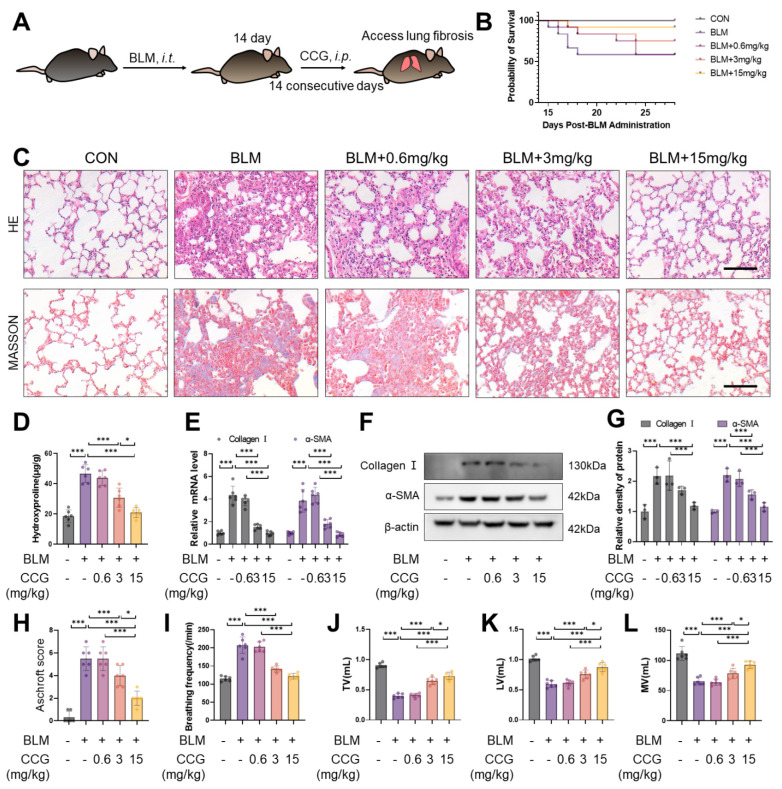
CCG mitigated BLM-induced pulmonary fibrosis. (**A**) Flowchart of CCG intervention protocol. (**B**) Survival rate of mice. (**C**) Lung morphology and ECM deposition examined via HE and Masson staining. Scale bars represent 100 μm. (**D**) Hydroxyproline contents in lung tissue homogenates. (**E**) mRNA levels of collagen I and α-SMA in mouse lungs assessed via qPCR. (**F**,**G**) Expression levels of collagen I and α-SMA detected using WB. (**H**) Pulmonary fibrosis score in mice. (**I**–**L**) Respiratory function tests, including breathing frequency, tidal volume (TV, mL), lung volume (LV, mL), and minute volume (MV, mL). Data represent means ± standard deviation, with each experiment independently repeated at least three times. (* *p* < 0.05, and *** *p* < 0.001).

Starting from the 14th day after BLM administration, the synthesis of extracellular matrix proteins, including collagen and myofibroblast differentiation, significantly increased [35]. Therefore, we initiated interventions by administering CCG via intraperitoneal injection (0.6 mg/kg, 3 mg/kg, 15 mg/kg) from day 14 and continued until day 28 (Figure 1A). The results indicate that CCG reduced the mortality rate of BLM-induced mice (Figure 1B,H). The HE and Masson staining results suggest that the thickening of alveolar walls and increased deposition of extracellular matrix induced by bleomycin were attenuated by CCG (Figure 1C). CCG alleviated the increase in hydroxyproline content (Figure 1D). Furthermore, the upregulation of mRNA and expression levels of Collagen 1 and α-SMA as markers of fibrosis induced by BLM were reversed by the concentration gradient of CCG (Figure 1E–G). Additionally, CCG improved the respiratory function of mice with pulmonary fibrosis (Figure 1I–L). These results suggest that CCG alleviates experimental mouse pulmonary fibrosis.

### 3.2. CCG Alleviated the Senescence of AECs and Fibroblasts Induced by BLM In Vivo

**Figure 2:**
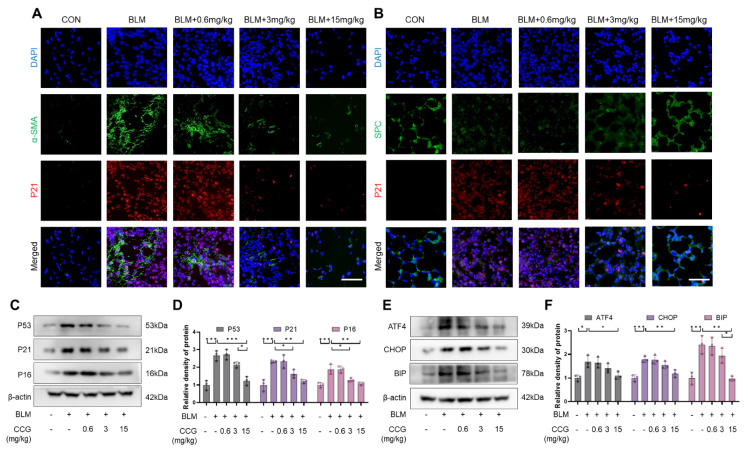
CCG alleviated BLM-induced pulmonary senescence. (**A**) Immunofluorescence staining showed the colocalization of the senescence marker P21 and the fibroblast marker α-SMA. Red represents P21, green represents α-SMA, and blue represents DAPI. Scale bars represent 100 μm. (**B**) Immunofluorescence staining showed the colocalization of the senescence marker P21 and the AEC marker SPC. Red represents P21, green represents SPC, and blue represents DAPI. Scale bars represent 100 μm. (**C**,**D**) Expression levels of senescence markers P53, P21, and P16 detected via WB. (**E**,**F**) Expression levels of ERS markers ATF4, CHOP, and BIP detected via WB. Data represent means ± standard deviation, with each experiment independently repeated at least three times. (* *p* < 0.05, ** *p* < 0.01, and *** *p* < 0.001).

Given the critical roles of AECs and fibroblast senescence in pulmonary fibrosis, the anti-senescence effect of CCG was observed. Immunofluorescence co-staining with P21 and SPC (AECs marker) [36] or α-SMA (fibroblast marker) [37] indicated that CCG reduced fibroblast senescence and decreased their activation into myofibroblasts. Similarly, CCG attenuated BLM-induced AECs senescence and damage (Figure 2A,B). Furthermore, we detected the expressions of senescence markers P53, P21, and P16 in whole lung tissue homogenates, revealing that CCG exhibited anti-senescence activity in lung tissues (Figure 2C,D). Additionally, the protein levels of ERS markers ATF4, CHOP, and BIP were reduced under CCG treatment (Figure 2E,F). These results suggest that CCG mitigates BLM-induced pulmonary senescence.

### 3.3. CCG Alleviated H_2_O_2_-Induced Senescence in AECs In Vitro

**Figure 3:**
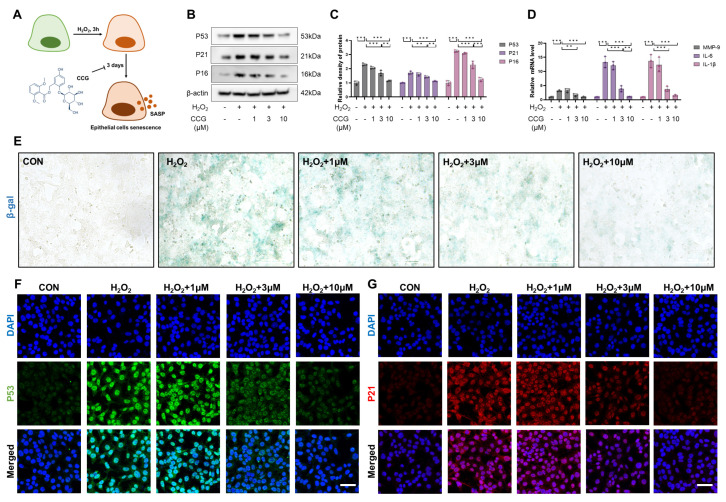
CCG reduced H_2_O_2_-induced AEC senescence. (**A**) Flowchart illustrating the intervention of CCG and H_2_O_2_ on AECs. (**B**,**C**) Expression levels of senescence markers P53, P21, and P16 detected via WB. (**D**) mRNA levels of SASP phenotype markers MMP-9, IL-6, and IL-1β detected via qPCR. (**E**) β-gal staining of AECs. Scale bars represent 100 μm. (**F**,**G**) Immunofluorescence staining reflecting the levels of senescence markers P53 and P21. Red represents P53, green represents P21, and blue represents DAPI. Scale bars represent 50 μm. Data represent means ± standard deviation, with each experiment independently repeated at least three times. (** *p* < 0.01, and *** *p* < 0.001).

H_2_O_2_ is the most common and widespread factor inducing senescence and was used to induce senescence in AECs (Figure 3A). The results indicate that treatment with CCG (1 μM, 3 μM, 10 μM) reduced the levels of senescence markers P53, P21, and P16 in a concentration-dependent manner (Figure 3B,C,F,G). Moreover, CCG was able to reverse the H_2_O_2_-induced SASP phenotype (Figure 3D). β-galactosidase staining suggested that CCG possessed potent anti-senescence activity (Figure 3E). These findings indicate that CCG can attenuate H_2_O_2_-induced senescence in AECs.

### 3.4. CCG Attenuated Natural Senescence in Primary Fibroblasts In Vitro

**Figure 4:**
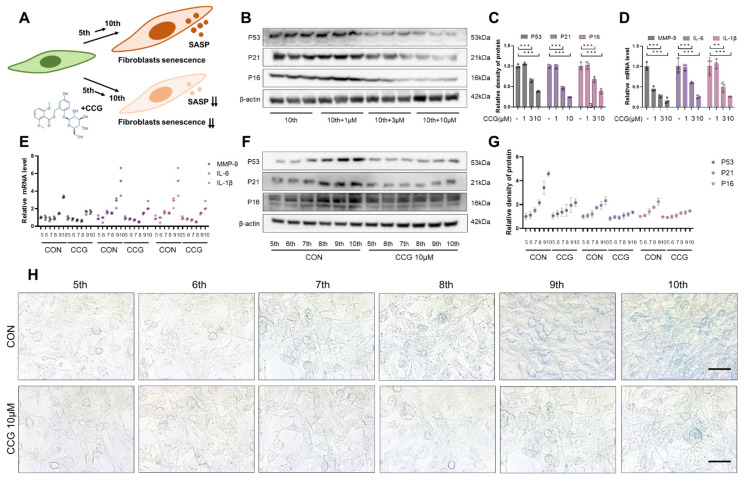
CCG delayed natural senescence of primary lung fibroblast. (**A**) Flowchart illustrating the intervention of CCG on primary fibroblasts. (**B**,**C**) WB analysis of the expression levels of senescence markers P53, P21, and P16 in the 10th passage primary fibroblasts treated with 1, 3, and 10 μM of CCG. (**D**) qPCR analysis of the mRNA levels of SASP phenotype markers MMP-9, IL-6, and IL-1β in the 10th passage primary fibroblasts treated with 1, 3, and 10 μM of CCG. (**E**) qPCR analysis of mRNA levels of MMP-9, IL-6, and IL-1β in the 5th-10th passage primary fibroblasts treated with 10 μM of CCG. (**F**,**G**) WB analysis of the protein levels of P53, P21, and P16 in the 5–10th passage primary fibroblasts treated with 10 μM of CCG. (**H**) β-gal staining of the 5–10th passage primary fibroblasts treated with 10 μM of CCG. Scale bars represent 100 μm. Data represent means ± standard deviation, with each experiment independently repeated at least three times. (** *p* < 0.01, and *** *p* < 0.001).

To ascertain whether CCG exhibits anti-senescence activity under different conditions, we selected a natural senescence model of primary fibroblasts (5th–10th) (Figure 4A). The results indicate that treatment with CCG (10 μM) delayed the natural senescence of primary fibroblasts (Figure 4B,C,H), while reducing the production of the fibroblast SASP phenotype (Figure 4D). Furthermore, the inhibitory effect of CCG on natural fibroblast senescence and SASP production showed a concentration-dependent trend (Figure 4E–G). These findings suggest that CCG can delay natural fibroblast senescence and SASP formation.

### 3.5. CCG Reduced ROS-Mediated ERS in AECs

**Figure 5:**
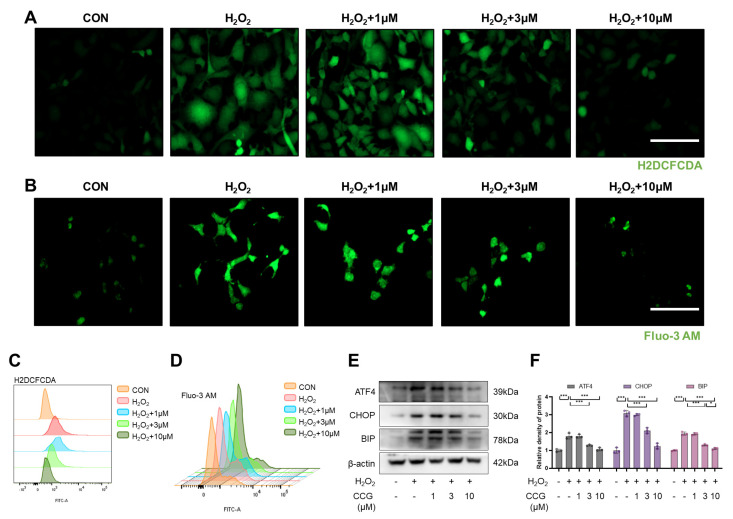
CCG attenuated ROS-mediated ERS in AECs. (**A**) H2DCFDA probe labeling of ROS (green) in AECs. Scale bars represent 100 μm. (**B**) Fluo-3AM probe (green) reflecting intracellular calcium influx in AECs. Scale bars represent 100 μm. (**C**) Flow cytometry detection of ROS levels in AECs. (**D**) Flow cytometry detection of intracellular calcium influx in AECs. (**E**,**F**) Western blot analysis of expression levels of ERS markers ATF4, CHOP, and BIP. Data represent means ± standard deviation, with each experiment independently repeated at least three times. (* *p* < 0.05, and *** *p* < 0.001).

ROS-mediated ERS is a major driving factor of senescence in AECs [38]. Therefore, we utilized the H2DCFCDA probe to label ROS. The results show that CCG reduced the ROS levels in AECs induced by H_2_O_2_ (Figure 5A,C). The Fluo-3AM probe was used to reflect the level of intracellular calcium influx, revealing that CCG reduced the H_2_O_2_-induced calcium influx (Figure 5B,D). Furthermore, the expression levels of key proteins involved in ERS, including ATF4, CHOP, and BIP, were decreased upon treatment with CCG (Figure 5E,F). Taken together, CCG attenuated ERS in AECs by reducing ROS levels.

### 3.6. CCG Alleviated ROS-Mediated ERS in Naturally Senescent Fibroblasts

**Figure 6:**
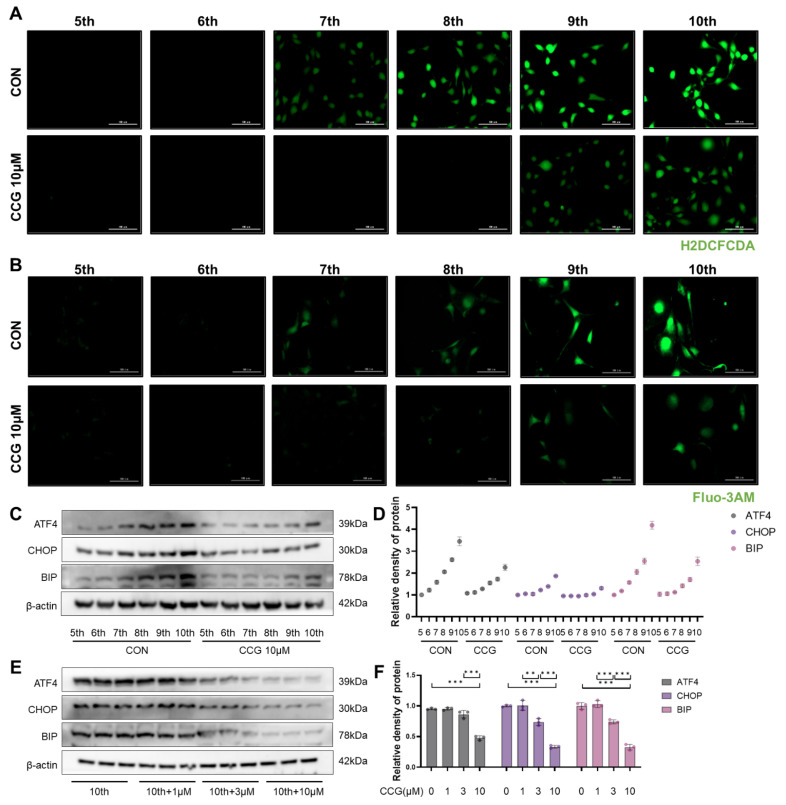
CCG attenuated ROS-mediated ERS in fibroblasts. (**A**) H2DCFDA probe labeling of ROS (green) in 5–10th passage fibroblasts. Scale bars represent 100 μm. (**B**) Fluo-3AM probe (green) reflecting intracellular calcium influx in 5–10th passage fibroblasts. Scale bars represent 100 μm. (**C**,**D**) Western blot analysis of expression levels of ERS markers ATF4, CHOP, and BIP in 5–10th passage fibroblasts after treatment with 10 μM CCG. (**E**,**F**) Western blot analysis of expression levels of ERS markers ATF4, CHOP, and BIP in 10th passage fibroblasts after treatment with 1, 3, and 10 μM CCG. Data represent means ± standard deviation, with each experiment independently repeated at least three times. (** *p* < 0.01, and *** *p* < 0.001).

Similarly, we observed the effects of CCG on ROS and ERS in naturally senescent fibroblasts. The results indicate that CCG reduced the levels of ROS in senescent fibroblasts (Figure 6A) as well as intracellular calcium influx (Figure 6B). Furthermore, CCG treatment decreased the levels of ATF4, CHOP, and BIP during the natural senescence process (Figure 6C,D) in a concentration-dependent manner (Figure 6E,F). These findings suggest that CCG alleviates ROS-mediated ERS in naturally senescent fibroblasts.

### 3.7. The SIRT1-P300 Signaling Pathway Mediated the Regulation of Antioxidant Enzymes through CCG

**Figure 7:**
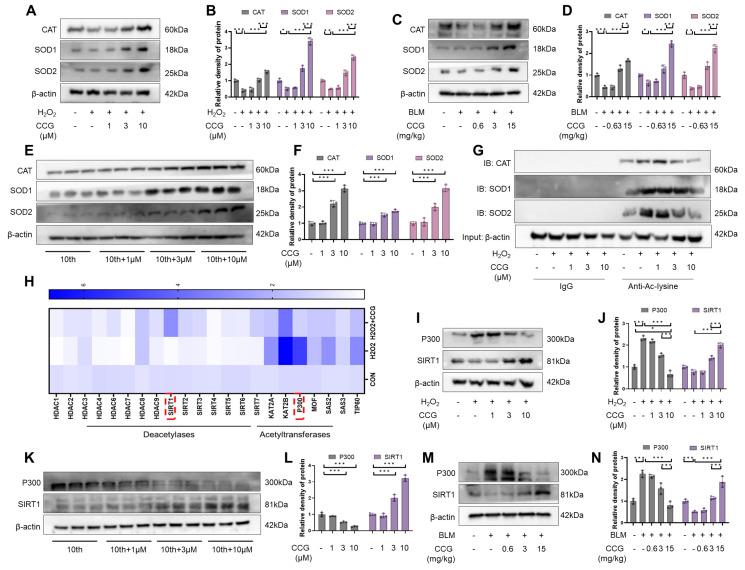
CCG regulated antioxidant enzyme expression via the SIRT1-P300 signaling pathway. (**A**,**B**) WB analysis of antioxidant enzyme CAT, SOD1, and SOD2 expressions in AECs. (**C**,**D**) WB analysis of CAT, SOD1, and SOD2 expressions in vivo. (**E**,**F**) WB analysis of CAT, SOD1, and SOD2 expressions in fibroblasts. (**G**) IP assay detecting the acetylation levels of CAT, SOD1, and SOD2. (**H**) qPCR analysis of mRNA levels of deacetylases and acetylases. (**I**,**J**) WB analysis of SIRT1 and P300 expressions in AECs. (**K**,**L**) WB analysis of CAT, SOD1, and SOD2 expressions in fibroblasts. (**M**,**N**) WB analysis of CAT, SOD1, and SOD2 expressions in vivo. Data represent means ± standard deviation, with each experiment independently repeated at least three times. (* *p* < 0.05, ** *p* < 0.01, and *** *p* < 0.001).

Due to the antioxidative effects of CCG, we hypothesized that CCG might reduce ROS levels by enhancing antioxidant enzymes. CAT, SOD, GPx, etc., are important antioxidant enzymes in cells playing crucial roles in scavenging reactive oxygen species and maintaining the redox balance within cells [39]. We observed a concentration-dependent promotion of CAT, SOD1, and SOD2 expressions through CCG in both AECs and fibroblasts (Figure 7A,B,E,F). This effect was consistent in the in vivo model treated with CCG (Figure 7C,D). The acetylation and deacetylation balance of intracellular antioxidant enzymes is a crucial step in their transcriptional regulation, with acetylation levels inversely correlated with antioxidant enzyme activity and expression. Therefore, we examined the acetylation levels of CAT, SOD1, and SOD2, and the results indicate that CCG treatment reduced the acetylation levels of antioxidant enzymes (Figure 7G). The balance of acetylation in cells is mainly regulated by acetyltransferases including KAT2A, KAT2B, P300, MOF, SAS2, SAS3, and TIP60 and deacetylases including the HDAC family and sirtuin family [40,41]. The qPCR results suggested that CCG significantly increased the level of SIRT1 and decreased the level of P300 in AECs (Figure 7H). Similarly, changes in the expression levels of SIRT1 and P300 were observed in fibroblasts, AECs, and in vivo under CCG treatment (Figure 7I–N). Overall, the SIRT1-P300 signaling pathway may be the main the mechanism through which CCG promotes the expressions of antioxidant enzymes.

### 3.8. CCG Promoted the Ubiquitination and Degradation of P300 via Trim72

**Figure 8:**
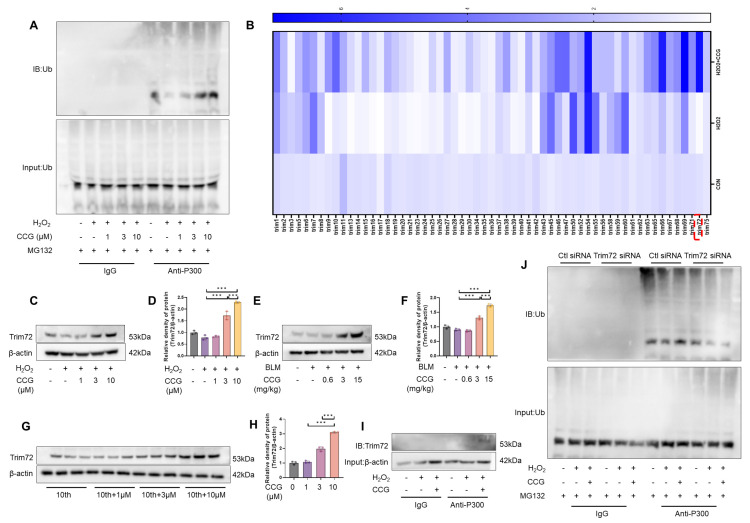
Trim72 mediated CCG-induced ubiquitination degradation of P300. (**A**) IP assay detecting the ubiquitination degradation levels of P300 in AECs. (**B**) qPCR analysis of mRNA levels of Trim family. (**C**,**D**) WB analysis of Trim72 expression in AECs. (**E**,**F**) WB analysis of Trim72 expression in vivo. (**G**,**H**) WB analysis of Trim72 expression in fibroblasts. (**I**) IP assay detecting the binding levels of Trim72 and P300. (**J**) IP assay detecting the ubiquitination degradation levels of P300 in AECs under Trim72 siRNA treatment. Data represent means ± standard deviation, with each experiment independently repeated at least three times. (*** *p* < 0.001).

In our study, we observed a significant reduction in P300 protein levels in AECs after treatment with CCG compared to the CON group (Figure 7I,J). This led us to speculate about a potential degradation mechanism for P300. The IP assay results show a significant increase in P300 ubiquitination levels under CCG treatment (Figure 8A). The Trim family is a group of proteins with ubiquitin ligase activity involved in regulating protein ubiquitination processes in cells [42]. Subsequently, we examined the regulation of Trim family mRNA levels by CCG and found that compared to the CON group, Trim72 expression decreased under H_2_O_2_ treatment, while it significantly increased under CCG treatment (Figure 8B). Consistent changes in Trim72 expression levels were observed in AECs, fibroblasts, and in vivo under CCG treatment (Figure 8C–H). Additionally, our study revealed for the first time that Trim72 can directly interact with P300 (Figure 8I). Following Trim72 siRNA treatment, we observed a reversal of the CCG-induced degradation of P300 (Figure 8J). Taken together, our findings suggest that Trim72 mediates the CCG-induced ubiquitination and degradation of P300.

### 3.9. SIRT1 Mediated the Anti-Senescence Effect of CCG in AECs

**Figure 9:**
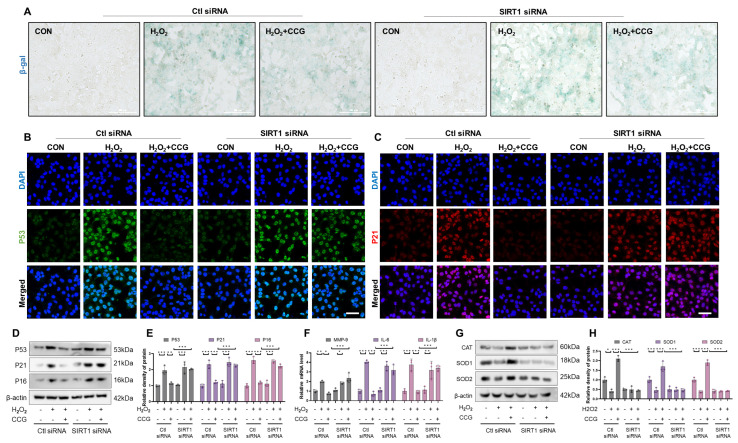
SIRT1 mediated the anti-senescence effect of CCG. (**A**) β-gal staining of AECs. Scale bars represent 100 μm. (**B**,**C**) Immunofluorescence staining reflecting the levels of the aging markers P53 and P21. Red represents P53, green represents P21, and blue represents DAPI. Scale bars represent 50 μm. (**D**,**E**) WB of the expression levels of the aging markers P53, P21, and P16. (**F**) qPCR analysis of mRNA levels of SASP phenotype markers MMP-9, IL-6, and IL-1β. (**G**,**H**) WB analysis of the expressions of antioxidant enzymes CAT, SOD1, and SOD2 in AECs. Data represent means ± standard deviation, with each experiment independently repeated at least three times. (* *p* < 0.05, ** *p* < 0.01, and *** *p* < 0.001).

SIRT1 is a NAD+-dependent deacetylase belonging to the Sirtuin family [43]. It participates in regulating various biological processes within the cell, including apoptosis, DNA repair, energy metabolism, and aging [44]. Under SIRT1 siRNA treatment, the regulatory effect of CCG on β-gal and the key senescence proteins P53, P21, and P16 was reversed (Figure 9A–E). Additionally, we observed that CCG’s inhibition of the SASP phenotype is also blocked after SIRT1 silencing (Figure 9F). Furthermore, the elevation of antioxidant enzyme levels induced by CCG treatment was reversed as well (Figure 9G,H). These results suggest that SIRT1 mediates the anti-senescence activity of CCG.

### 3.10. SIRT1 Mediated the Effect of CCG on ROS-Mediated ERS in AECs

**Figure 10:**
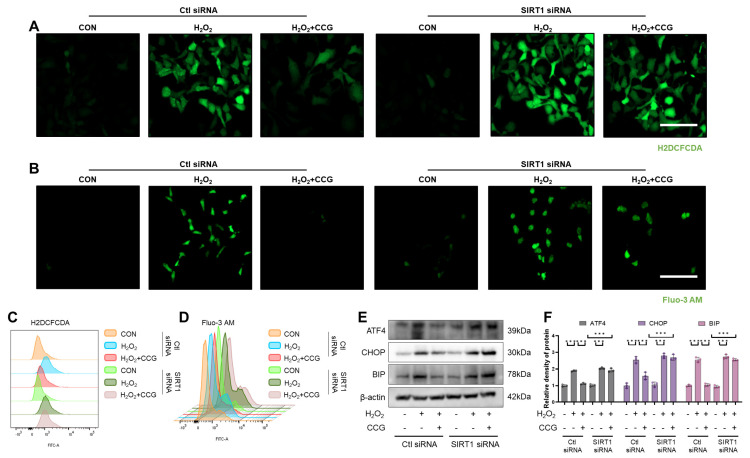
SIRT1 mediated the effect of CCG on ROS-mediated ERS in AECs. (**A**) H2DCFCDA probe labeling of ROS (green) in AECs. Scale bars represent 100 μm. (**B**) Fluo-3AM probe (green) reflecting intracellular calcium influx in AECs. Scale bars represent 100 μm. (**C**) Flow cytometry analysis of ROS levels in AECs. (**D**) Flow cytometry analysis of intracellular calcium influx in AECs. (**E**,**F**) WB analysis of expression levels of ERS markers ATF4, CHOP, and BIP. Data represent means ± standard deviation, with each experiment independently repeated at least three times. (*** *p* < 0.001).

In SIRT1 siRNA-treated AECs, we observed a reversal of CCG’s reduction in ROS levels induced by H_2_O_2_ (see Figure 10A). Additionally, the results indicate that SIRT1 silencing blocked CCG’s inhibition of ROS-mediated calcium influx and the suppression of ATF4, CHOP, and BIP levels (see Figure 10B–F). Overall, SIRT1 regulates CCG’s inhibition of ROS-mediated ERS in AECs.

### 3.11. CCG Inhibited Lung Aging in Progeroid Mice

**Figure 11:**
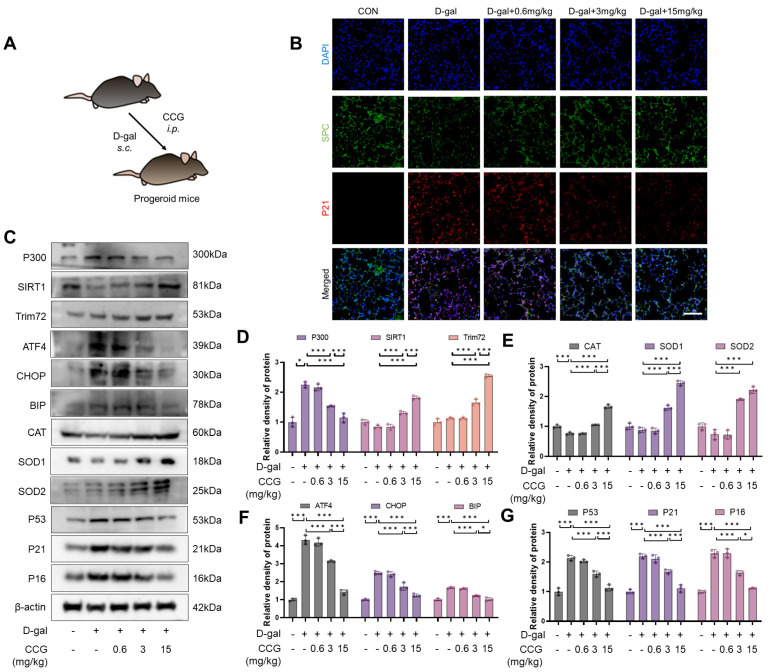
CCG alleviated D-gal-induced lung senescence in mice. (**A**) Flowchart of CCG intervention. (**B**) Immunofluorescence staining showing the co-staining of the senescence marker P21 and the AEC marker SPC. Red represents P21, green represents SPC, and blue represents DAPI. Scale bars represent 100 μm. (**C**–**G**) WB of the expression levels of P300, SIRT1, Trim72, CAT, SOD1, SOD2, P53, P21, and P16. Data represent means ± standard deviation, with each experiment independently repeated at least three times. (* *p* < 0.05, and *** *p* < 0.001).

D-gal is widely used to induce premature aging in mouse models [45,46]. To validate the direct anti-lung aging activity and molecular mechanism of CCG, we employed a D-galactose-induced premature aging model (Figure 11A). The immunofluorescence results indicate that CCG treatment reversed D-gal-induced lung epithelial cell damage and aging (Figure 11B). The results show that in lung tissue homogenates, CCG treatment reduced the levels of ATF, CHOP, BIP, P53, P21, and P16, while increasing the levels of CAT, SOD1, and SOD2. This may be closely associated with the upregulation of TRIM72, SIRT1 levels and downregulation of P300 levels following CCG treatment (Figure 11C–G).

## 4. Discussion

Our study revealed for the first time that CCG upregulates the expression of SIRT1 and downregulates the expression of P300. Meanwhile, through the Trim72-mediated ubiquitination and degradation of P300, CCG reduces the acetylation levels of downstream antioxidant enzymes while promoting their expressions. This cascade of effects inhibits ROS-mediated ERS and prevents H_2_O_2_-induced senescence in AECs as well as natural senescence in primary fibroblasts. Consequently, CCG demonstrates significant efficacy in inhibiting BLM-induced pulmonary fibrosis and lung senescence. Furthermore, we validated the anti-senescence activity of CCG and its mechanism in a D-gal-induced progeroid senescence model.

IPF is a chronic progressive disease with a highly malignant nature, with a median survival time of 2–3 years after diagnosis [8]. While various factors increasing the risk of IPF development have been reported, its most prominent risk factor is aging [47,48,49]. Studies have shown dramatic increases in the prevalence and incidence of IPF with advancing age, with individuals over 70 having a 6.9-fold higher risk of developing IPF compared to those over 40 [50]. Therefore, IPF can be described as a disease closely associated with aging. The senescent state of AECs and lung fibroblasts, as the primary cells maintaining lung function, is intricately linked to the occurrence and development of pulmonary fibrosis [9,10]. Multiple genome-wide screening studies on IPF have indicated that dysfunction and/or reduction in the number of AECs and cellular senescence are key factors in the pathogenesis of IPF [51]. Even for one of the identified top genetic variants of IPF, the telomere-related gene Tert, its deletion in mice still requires exposure to LPS or BLM treatment in the third generation of mice to enhance fibrosis [52]. Endogenous substances such as spermidine and EETs have been proven to inhibit AEC senescence and thereby suppress pulmonary fibrosis [12,19]. Fibroblasts, as another major cell population in the lungs, are believed to undergo increased and sustained senescence in IPF lungs, consistent with observations in aged mice induced with bleomycin [53,54]. Senescent fibroblasts secrete the SASP, releasing large amounts of pro-inflammatory cytokines, growth factors, etc., causing sustained low-grade inflammation in the surrounding microenvironment, thereby triggering more severe fibrosis [55]. Therefore, there is an urgent need to research specific drugs that inhibit the senescence of AECs and fibroblasts. In our study, CCG exhibited inhibitory effects on both exogenous senescence (e.g., H_2_O_2_-induced AECs) and endogenous senescence (e.g., naturally aged fibroblasts) in vitro, and inhibited BLM-induced lung senescence and D-gal-induced lung senescence in vivo, suggesting that CCG may be a potent anti-senescence agent with potential. The inducers of aging are diverse and complex. This study has yet to observe whether CCG can counteract cell aging induced by various exogenous stimuli, such as X-ray and BLM. Therefore, further research is warranted to investigate the broad anti-aging effects of CCG.

During the process of senescence in AECs and fibroblasts, they are the main cells that are both exposed to and producing a large amount of ROS in the microenvironment [56]. ROS can mediate ERS, serving as both a driving factor for senescence phenomena and a consequence of the senescent phenotype [57]. As shown to be consistent with other studies, treatment with CCG reduced ROS levels in H_2_O_2_-induced AECs and alleviated ERS. It also alleviated ROS levels and ERS in the 5–10th passage fibroblasts. Additionally, it has been reported that the redox system is impaired in senescent cells, especially the deficiency of antioxidant enzymes. In our study, we found that CCG promotes the expressions of CAT, SOD1, and SOD2 both in vitro and in vivo. Therefore, we believed that CCG alleviates endogenous or exogenous ROS-mediated ERS by promoting the expressions of antioxidant enzymes.

In the progression of lung senescence and/or fibrosis, the acetylation modification of antioxidant enzymes is a crucial step in regulating antioxidant enzyme activity and expression [58]. Antioxidant enzymes in senescent lungs exhibit elevated levels of acetylation. The restoration of EC-SOD activity in senescent lung fibroblasts through HDAC-mediated deacetylation resists senescence [59]. In our study, CCG treatment was found to decrease the acetylation levels of CAT, SOD1, and SOD2. Thus, we hypothesize that the activity of CCG may be associated with modulating the acetylation balance of antioxidant enzymes. Therefore, in AECs treated with CCG, we primarily examined the levels of acetyltransferases and deacetylases. The results indicate a significant increase in SIRT1 levels and a significant decrease in P300 levels. SIRT1 is a deacetylase involved in regulating the cell lifecycle, metabolism, and stress response [44]. P300 is a histone acetyltransferase involved in gene transcription, cell proliferation, and differentiation [28]. Under the action of SIRT1 siRNA, the effects of CCG on ERS and senescence were reversed. Thus, we believe that the acetylation balance regulated by SIRT1-P300 may be the main signaling pathway through which CCG exerts its anti-senescence activity. However, given the direct effects of SIRT1-P300 on senescence genes P53, P21, and P16, the regulation of antioxidant enzymes by SIRT1-P300 reflects only a partial mechanism of CCG’s anti-senescence activity, which still requires further investigation. Furthermore, CCG has been reported to regulate the Nrf2 signaling pathway in osteoclasts and the AKT signaling pathway in osteoblasts, thereby exerting antioxidant activity [60,61]. The SIRT1 pathway has been confirmed to regulate the Nrf2 signaling pathway [62,63]. As one of the upstream proteins of Nrf2, SIRT1 promotes Nrf2 phosphorylation by deacetylating Nrf2, and it indirectly affects the activity and function of Nrf2 by regulating other signaling pathways such as AMPK, FOXO, and AKT, thereby participating in mediating the Nrf2-mediated antioxidant stress response [27,64]. Moreover, there is a close interaction between SIRT1 and AKT [65]. The deacetylation mediated by SIRT1 regulates the binding of AKT to PIP3, activating AKT [66]. On the other hand, the activity of AKT can cooperatively regulate the nuclear localization of FOXO3A and the activity of eNOS with SIRT1, affecting the occurrence of oxidative stress and aging [65,67]. Therefore, we believe that the antioxidant and anti-aging effects of CCG may be mediated by SIRT1, but there may be multiple downstream signaling pathways involved, which still need further research to confirm. Additionally, we found a ubiquitination degradation pathway for P300. Trim72, as a ubiquitin ligase, has been shown to protect against fibrosis when upregulated [68]. CCG treatment significantly upregulates its levels. Under Trim72 siRNA treatment, the CCG-induced ubiquitination degradation of P300 was reversed, and co-IP experiments suggested that Trim72 may directly bind to P300. Therefore, we speculate that Trim72 mediates the degradation of P300 by directly binding to it. This study is the first to reveal the mechanism of action between Trim72 and P300, suggesting that Trim72’s anti-fibrotic effect may be related to P300. However, our study has not thoroughly explored how CCG regulates Trim72, which may be a result of GSK3β phosphorylation or may be related to the activity or expression of SIRT1. Therefore, further investigation is needed to explore the regulatory mechanism of Trim72.

In our study, we also observed the regulation of GSK3β through CCG. The activity of GSK3β is closely associated with pulmonary fibrosis and lung aging, where the phosphorylation of GSK3β at Ser21 and Ser9 leads to decreased activity, thereby inhibiting pulmonary fibrosis and lung aging [69,70]. Our experiments suggest that CCG can promote its phosphorylation at the Ser9 site (Supplementary Figures S3 and S4). Phosphorylated GSK3β plays a crucial role in regulating SIRT1 and P300. Phosphorylated GSK3β not only regulates the phosphorylation status of SIRT1, affecting its activity, but also activates the Wnt/β-catenin signaling pathway to promote the expression of SIRT1 [71,72,73]. Additionally, phosphorylated GSK3β can alter the structure or affinity of P300, thereby regulating its activity [74]. Additionally, the SIRT1-P300 signaling pathway has been reported to alter the acetylation levels of GSK3β, thereby regulating its activity [75,76]. Moreover, the phosphorylation level of GSK3β is regulated by SIRT1 [77]. Therefore, further experimental research is needed to elucidate the upstream and downstream relationships between CCG-mediated GSK3β and the SIRT1-P300 signaling pathway.

## 5. Conclusions

In summary, our data demonstrate that CCG serves as a potent anti-aging and anti-fibrotic agent. By modulating downstream SIRT1-P300 signaling, regulating the expression of antioxidant enzymes, and inhibiting ROS-mediated ER stress, CCG exerts its anti-aging and anti-fibrotic effects. Additionally, our study provides the first evidence of Trim72’s role in regulating P300 ubiquitination, which may suggest a novel mechanism for the Trim72-mediated modulation of pulmonary fibrosis.

## Figures and Tables

**Table 1:** The information of the primary antibodies.

Antibodies	Source	Dilution Ratio
Anti-Collagen Ⅰ Polyclonal antibody	Abcam	1:5000
Anti-α-SMA Polyclonal antibody	Proteintech	1:4000
Anti-P53 Monoclonal antibody	Proteintech	1:10,000
Anti-P21 Polyclonal antibody	Abcam	1:5000
Anti-P16 Polyclonal antibody	Abcam	1:5000
Anti-ATF4 Polyclonal antibody	Proteintech	1:1000
Anti-CHOP Polyclonal antibody	Proteintech	1:1000
Anti-BIP Polyclonal antibody	Proteintech	1:6000
Anti-Catalase Polyclonal antibody	Abcam	1:2000
Anti-Superoxide Dismutase 1 Polyclonal antibody	Abcam	1:20,000
Anti-SOD2 Polyclonal antibody	Abcam	1:2000
Anti-P300 Polyclonal antibody	Zen-Bio	1:1000
Anti-SIRT1 Polyclonal antibody	Proteintech	1:2000
Anti-Ubiquitin Polyclonal antibody	Abcam	1:2000
Anti-Trim72 Polyclonal antibody	Proteintech	1:5000
Anti-GSK3β Polyclonal antibody	Proteintech	1:4000
Anti-Phospho-GSK3β Monoclonal antibody	Proteintech	1:5000
Anti-β-Actin Recombinant antibody	Proteintech	1:20,000

**Table 2:** The information of the qPCR sequences.

Name	Forward Primer (5′–3′)	Reverse Primer (5′–3′)
m-Colα1	*ATGTTCAGCTTTGTGGACCTC*	*CTGTACGCAGGTGATTGGTG*
m-α-sma	*TGGCTATTCAGGCTGTGCTGTC*	*CAATCTCACGCTCGGCAGTAGT*
m-MMP-9	*AGACGCCCATTTCGACGATGAC*	*CAAACCGAGTTGGAACCACGAC*
m-IL-6	*CCAGGAGCCCAGCTATGAAC*	*CCCAGGGAGAAGGCAACTG*
m-IL-1β	*CAGGCAGGCAGTATCACTCA*	*AGCTCATATGGGTCCGACAG*
m-Trim1	*AGTTGTTTGAAGACCCCCTTCT*	*TGTAGGACACTGGAAAGCAGTAA*
m-Trim2	*TGGACAGTTCAAAAGTCGTTTCG*	*AATGCTAACCCACTTGTTGTCAT*
m-Trim3	*GCGTCTCAGCCCTACAAAACA*	*AAACTCCATTGTCTTGCCTTCA*
m-Trim5	*AAGAAAGTTCCGAGCCCCTG*	*GTAGCGTTGAGCCTCTGTGA*
m-Trim6	*ATGACTTCAACAGTCTTGGTGG*	*TTCCCAGGCTGATAGGAGGTC*
m-Trim7	*ACAGAAACAGAATGAGAACCTGG*	*GCTCAGTGTGCTTTTGAACTCC*
m-Trim8	*AGGGACACTCGGTGTGTGA*	*TGTCTGCCGCAAGTCTTCATC*
m-Trim9	*CTTGGGCAATAACTGAAGGAGG*	*GCTGGAGTAGAAGTCGGGG*
m-Trim10	*GGAACACGGGGAGAAAATCTAC*	*AGACACACGAGACACTTCTGT*
m-Trim11	*GCCCTCATCTCCGAGCTTG*	*CGCAGCACTCAATGCAGAG*
m-Trim13	*TGATGACCCCCGAGTGTTG*	*TTTCCTTACGGCAGGTAGGAC*
m-Trim14	*GTGCGTGTGCAGAAGCTAATC*	*CTGCGTAAACCTTGAGCCTTT*
m-Trim15	*CCTGAGCGAGACCTACTGTGA*	*AGAGCTTCTAACCGACTCCTG*
m-Trim16	*TCTTGGGGCCAGCAGAGTAA*	*CTCACAGTAGTTCACCATGCAG*
m-Trim17	*CTTGCCAGACGGTTACAAGAG*	*CTCAGCCACTTTTGTCAGGAG*
m-Trim18	*CTGTGACGGCACCTGTCTC*	*AAACGGCTGACTGTTGGTCTT*
m-Trim19	*CAGGCCCTAGAGCTGTCTAAG*	*ATACACTGGTACAGGGTGTGC*
m-Trim20	*TCATCTGCTAAACACCCTGGA*	*GGGATCTTAGAGTGGCCCTTC*
m-Trim21	*GGGAGGAGGTCACCTGTTCTA*	*CATTACCGTGTTCTTTTGCAGC*
m-Trim23	*ACCAGAAGCTAATCAGATCCGA*	*TGGCTCACAGTCAAACTGCTG*
m-Trim24	*TCAACAGGCCATAAAACAGTGG*	*GGCACTCGGGACATGAACTG*
m-Trim25	*GATGAGACGTGGGTCGTCC*	*TCTGTGTGAGCCATTCCAATTC*
m-Trim26	*TCGGCCAGTGGATACCTACAT*	*CTGCACTTGTGATTGTGGGG*
m-Trim27	*GGAGCAAATCCAGAACCGACT*	*GCCCCGTTGATGCTGTTATAG*
m-Trim28	*CGGCGCTATGGTGGATTGT*	*GGTTAGCATCCTGGGAATCAGAA*
m-Trim29	*AGAATGGCACTAAAGCAGACAG*	*AAATAGGCCACTCTTCCCCTC*
m-Trim30	*CTGTGAGTGCTGATTGTAACCA*	*ACTCGGCATACAGGGCAGT*
m-Trim34	*GTAATAACGGTATCTTGGGCTCC*	*TGCGTTGTCTAACATCAAACCTT*
m-Trim35	*TTCCGGGCCAAGTGTAAGAAC*	*CCAAGTCGTTTGCACCTCA*
m-Trim36	*GGCTACATTATGGAATTGCTTGC*	*GGATCAGCGGGTGGGTAAAC*
m-Trim37	*TCCAAGCTCTGTTGTTTCAGC*	*TTCCGCCCAACGACAGTTC*
m-Trim38	*ATGGGCTCAGACTTTAGCACG*	*CTGTTTTTGGGCTGACATTGC*
m-Trim39	*AACAGCTAATTGCGGATGTGA*	*ACAAACTTGACGCTTTTCCGAT*
m-Trim40	*TCATCTGCTGGTCTTCTCCC*	*CAGGAGCTCCAAACCCCAAT*
m-Trim41	*ATGAGCCGCATGTTTTGTCAG*	*GCCCCTAGTACACAGCAGT*
m-Trim42	*ATGGAGACGGCTATGTGTGTC*	*GCACTTACAGTTGGGGTCATT*
m-Trim43	*TGAAGGACTATAGGCGGTGGA*	*AGTGTTCACGTCCTATGCGG*
m-Trim45	*TCAGGCAAGACTCATTGTCCT*	*ACGGATGTCCACTACTGAGAAT*
m-Trim46	*GGTGAGGATATGCAGACCTTCA*	*TTGTGGGTACAAGGCAGCAC*
m-Trim47	*GGTGAGCCAGATGTTTGCC*	*TCCCTCTTCGATGAACCCCAT*
m-Trim50	*CCCATTTGCCTGGAGGTCTTC*	*CAGGACAGCATAGCTCGGAG*
m-Trim52	*ATGCAGTCACTTCGGGAAGAA*	*CTATGGCTATGACCGACCCAC*
m-Trim54	*GGAGAAGCAGCTCATTTGCC*	*CCTCCTGAAGACACCGTTGTG*
m-Trim55	*AAAGCAACTGATCTGTCCCAT*	*TGTGGGTAAGTACGGGTTAGAG*
m-Trim56	*CAGCGATTTCCTAGCCTGTAAA*	*GACCACCGATGTCCAGTTGT*
m-Trim58	*AGTGGGACTGATGAGTGGGT*	*AATGAAGCCTCGGGCAGTAG*
m-Trim59	*ATGCACAATTTTGAGGAGGAGT*	*GCAGTTAGGACACTTGAGTGGAA*
m-Trim60	*GCACAACTTCTGTTTTGCCTG*	*CAGTCATGTTACGGAACTGGTAG*
m-Trim61	*CATCTTGCCCCCTGAAAGAAC*	*GGTCAGCATCAGCGGATCAC*
m-Trim62	*CTTCGAGGAGTTGCAGAGAGA*	*GGCGTGAACATAATGCGGTC*
m-Trim63	*GTGTGAGGTGCCTACTTGCTC*	*CTGCTCGCAGTAGATGCTCA*
m-Trim65	*GAGGACGTGGTGACTTGCTC*	*GCTAGGCATGGGGTTTCGAT*
m-Trim66	*CTTTGCCTTGTACTGCCCTCT*	*TTTTCCACGGGCCAAACAAAG*
m-Trim67	*CCACTCTCTGCGAGCAATG*	*GCAGGCTCTTGGTAGAGGAC*
m-Trim68	*TCCCAGAACTTGAGCTACACC*	*GCTCAGTCTTCTGTCCTTGGA*
m-Trim69	*AACCACCACCCATTTACCCTC*	*ACGCCATGAATCCTGGATGC*
m-Trim71	*CAAGCTGGAGAGCACCATCA*	*TGGATTTCTTATGTGCCACCTG*
m-Trim72	*CCGCAGGCTCTAAGCACTAAC*	*GGTGGCTGAACTAGCCGAT*
m-Trim75	*TTGGGTACCAACTGTCAGCC*	*AGACGGACCTTGTCTACAACA*
m-β-actin	*TTCCAGCCTTCCTTCTTG*	*GGGAGCCAGAGCAGTAATC*
m-HDAC1	*AGTCTGTTACTACTACGACGGG*	*TGAGCAGCAAATTGTGAGTCAT*
m-HDAC2	*GGAGGAGGCTACACAATCCG*	*TCTGGAGTGTTCTGGTTTGTCA*
m-HDAC3	*GCCAAGACCGTGGCGTATT*	*GTCCAGCTCCATAGTGGAAGT*
m-HDAC4	*CTGCAAGTGGCCCCTACAG*	*CTGCTCATGTTGACGCTGGA*
m-HDAC5	*TGCAGCACGTTTTGCTCCT*	*GACAGCTCCCCAGTTTTGGT*
m-HDAC6	*TCCACCGGCCAAGATTCTTC*	*CAGCACACTTCTTTCCACCAC*
m-HDAC7	*GGCAGGCTTACACCAGCAA*	*TGGGCAGGCTGTAGGGAATA*
m-HDAC8	*ACTATTGCCGGAGATCCAATGT*	*CCTCCTAAAATCAGAGTTGCCAG*
m-SIRT1	*GCTGACGACTTCGACGACG*	*TCGGTCAACAGGAGGTTGTCT*
m-SIRT2	*GCCTGGGTTCCCAAAAGGAG*	*GAGCGGAAGTCAGGGATACC*
m-SIRT3	*ATCCCGGACTTCAGATCCCC*	*CAACATGAAAAAGGGCTTGGG*
m-SIRT4	*GTGGAAGAATAAGAATGAGCGG A*	*GGCACAAATAACCCCGAGG*
m-SIRT5	*CTCCGGGCCGATTCATTTCC*	*GCGTTCGCAAAACACTTCCG*
m-SIRT6	*ATGTCGGTGAATTATGCAGCA*	*GCTGGAGGACTGCCACATTA*
m-SIRT7	*CAGGTGTCACGCATCCTGAG*	*GCCCGTGTAGACAACCAAGT*
m-KAT2A	*CGAGTTGTGCCGTAGCTGTGA*	*ACCATTCCCAAGAGCCGGTTA*
m-KAT2B	*GAAGCCGCCATTTGAGAAGC*	*AGTTGATGCGGTTCAGAAACA*
m-P300	*GCCCGTGTAGACAACCAAGT*	*GCCCGTGTAGACAACCAAGT*
m-MOF	*CTGGAAGGGCCAGCATGTTA*	*GGTTAGAGGCCAGGAAACCC*
m-SAS2	*TTCGGCTCGCTGCTCATCC*	*GACTCTGCTCCCTCGCCAC*
m-SAS3	*GCAGTCTCACCCAGACCACC*	*CAAAATGACAGCCGAAATTG*
m-TIP60	*GGCTGGACTTAAAGA AGAT*	*GGACTTAAAGAAGATCCAA*

## Data Availability

The data is contained within the article and Supplementary Materials.

## Supplementary Materials

The following supporting information can be downloaded at: https://www.mdpi.com/article/10.3390/antiox13040420/s1, Supplementary Figure S1. Identification of primary fibroblasts. Immunofluorescence staining reflecting the expression levels of Vimentin in the primary mouse fibroblasts. Red represents Pho-Vimentin, and blue represents DAPI. Scale bars represent 100 μm. Supplementary Figure S2. The HPLC spectrum of CCG. Supplementary Figure S3. CCG promoted the phosphorylation of GSK3β. (A,B) Network pharmacology screening of CCG target proteins. (C) CESTA reflected the binding of CCG to GSK3β. (D,E) WB analysis of the protein levels of Pho-GSK3β and GSK3β in fibroblasts. (F,G) WB analysis of the protein levels of Pho-GSK3β and GSK3β in AECs. (H) Immunofluorescence staining showing the levels of Pho-GSK3β and P21 in AECs. Red represents P21, green represents Pho-GSK3β, and blue represents DAPI. Scale bars represented 50 μm. (I,J) WB analysis of the protein levels of Pho-GSK3β and GSK3β in vivo. Data represent means ± standard deviation, with each experiment independently repeated at least three times. (*** *p* < 0.001). Supplementary Figure S4. CCG promoted the phosphorylation of GSK3β in in the lungs of D-gal-induced mice. (A) Immunofluorescence staining reflecting the expression levels of Pho-GSK3β in the lungs. Green represents Pho-GSK3β, and blue represents DAPI. Scale bars represent 100 μm. (B,C) WB analysis of the protein levels of Pho-GSK3β and GSK3β in vivo. Data represent means ± standard deviation, with each experiment independently repeated at least three times. (* *p* < 0.05, and *** *p* < 0.001).

## Abbreviations

CCG, Curculigoside; IPF, idiopathic pulmonary fibrosis; AECs, alveolar epithelial cells; ERS, endoplasmic reticulum stress; D-gal, D-galactose; β-gal, β-galactosidase; DMSO, dimethyl sulfoxide; *i.t.*, intratracheal injection; *i.p.*, intraperitoneal injection; *s.c.*, subcutaneous; Trim72, tripartite motif-containing protein 72; GSK3β, glycogen Synthase Kinase 3 β; SIRT1, sirtuin 1; α-SMA, α-smooth muscle actin; ATF4, activating transcription factor 4; CHOP, C/EBP homologous protein; BIP, binding immunoglobulin protein; ROS, reactive oxygen species; H_2_O_2_, hydrogen peroxide; SASP, senescence-associated secretory phenotype; BLM, bleomycin; CAT, catalase; SOD1, superoxide dismutase 1; SOD2, superoxide dismutase 2; BALF, bronchoalveolar lavage fluid.
