# Supplementary material for: Curculigoside Attenuates Endoplasmic Reticulum Stress-Induced Epithelial Cell and Fibroblast Senescence by Regulating the SIRT1-P300 Signaling Pathway
Source: Antioxidants (Basel). 2024 Mar 29;13(4):420. doi: 10.3390/antiox13040420 (PMC11047561; doi:10.3390/antiox13040420)
Supplement: Supplementary file 1 [file antioxidants-13-00420-s001.zip › antioxidants-2928994-supplementary.pdf]

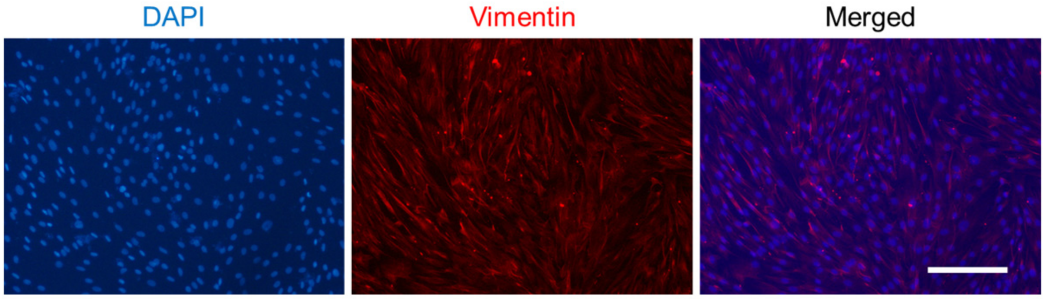

Supplementary Figure S1. Identification of primary fibroblasts. Immunofluorescence staining reflecting the expression levels of Vimentin in the primary mouse fibroblasts. Red represents Pho-Vimentin, and blue represents DAPI. Scale bars represent 100 μm.

```
Acq. Operator   : SYSTEM                          Seq. Line :    7
Acq. Instrument : 1260lc                           Location : Vial 34
                                                        Inj :    1
                                                 Inj Volume : 2.000 µl
```

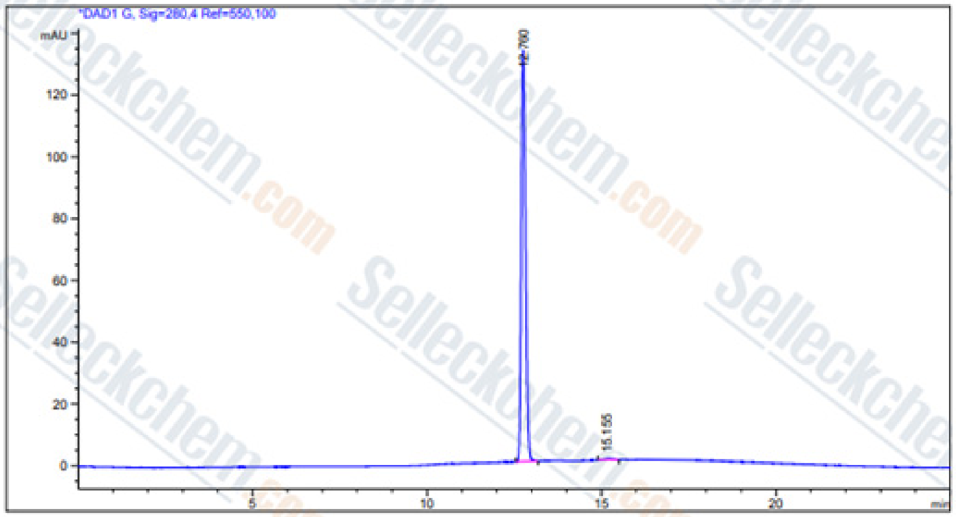

Area Percent Report

```
Sorted By             :      Signal
Multiplier            :      1.0000
Dilution              :      1.0000
Use Multiplier & Dilution Factor with ISTDs


Signal 1: DAD1 G, Sig=280,4 Ref=550,100
 Signal has been modified after loading from rawdata file!
```

Peak #	RetTime [min]	Type	Width [min]	Area [mAU*s]	Height [mAU]	Area %
1	12.760	BB	0.1326	1138.42017	133.34436	99.8459
2	15.155	BB	0.2102	1.75701	0.36703	0.1541
Totals :				1140.17718	134.51139	

*** End of Report ***

Supplementary Figure S2. The HPLC spectrum of CCG.

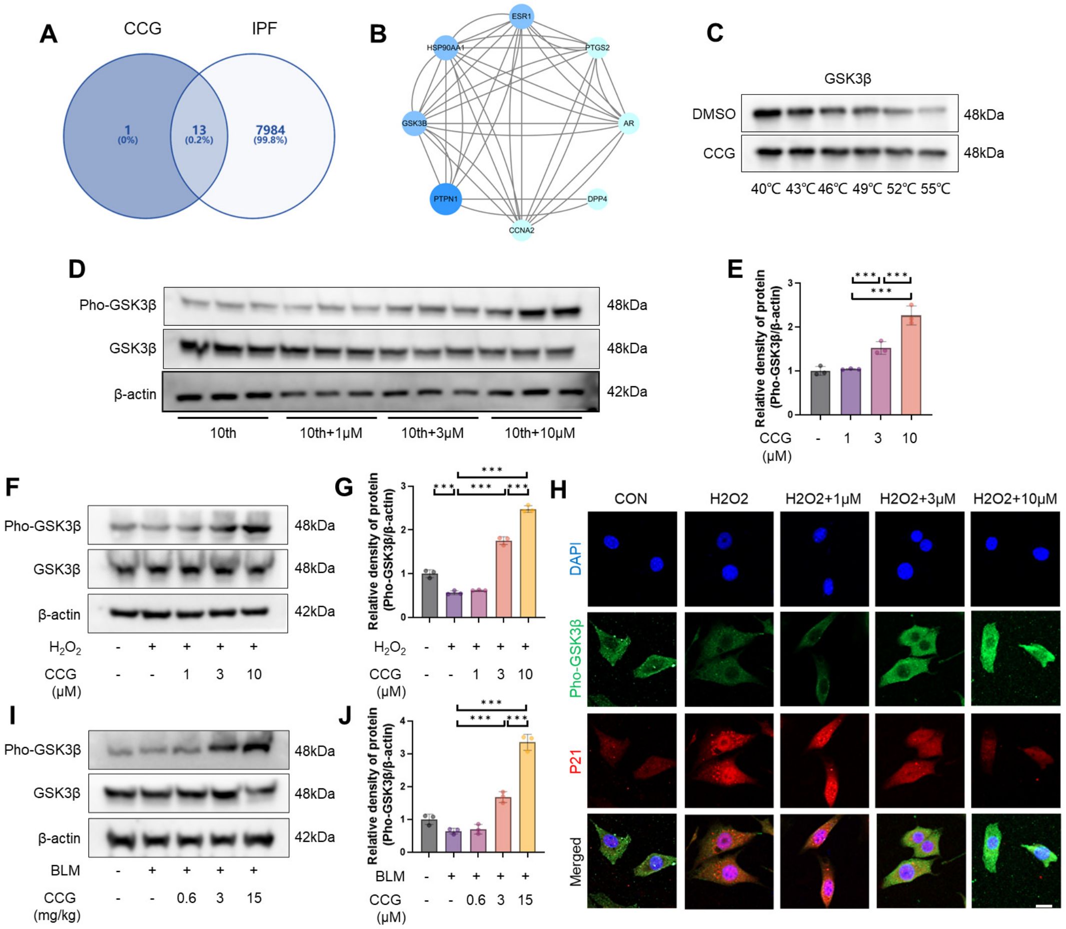

Supplementary Figure S3. CCG promoted the phosphorylation of GSK3β. (A,B) Network pharmacology screening of CCG target proteins. (C) CESTA reflected the binding of CCG to GSK3β. (D,E) WB analysis of the protein levels of Pho-GSK3β and GSK3β in fibroblasts. (F,G) WB analysis of the protein levels of Pho-GSK3β and GSK3β in AECs. (H) Immunofluorescence staining showing the levels of Pho-GSK3β and P21 in AECs. Red represents P21, green represents Pho-GSK3β, and blue represents DAPI. Scale bars represented 50 μm. (I,J) WB analysis of the protein levels of Pho-GSK3β and GSK3β in vivo. Data represent means ± standard deviation, with each experiment independently repeated at least three times. (*** $p < 0.001$).

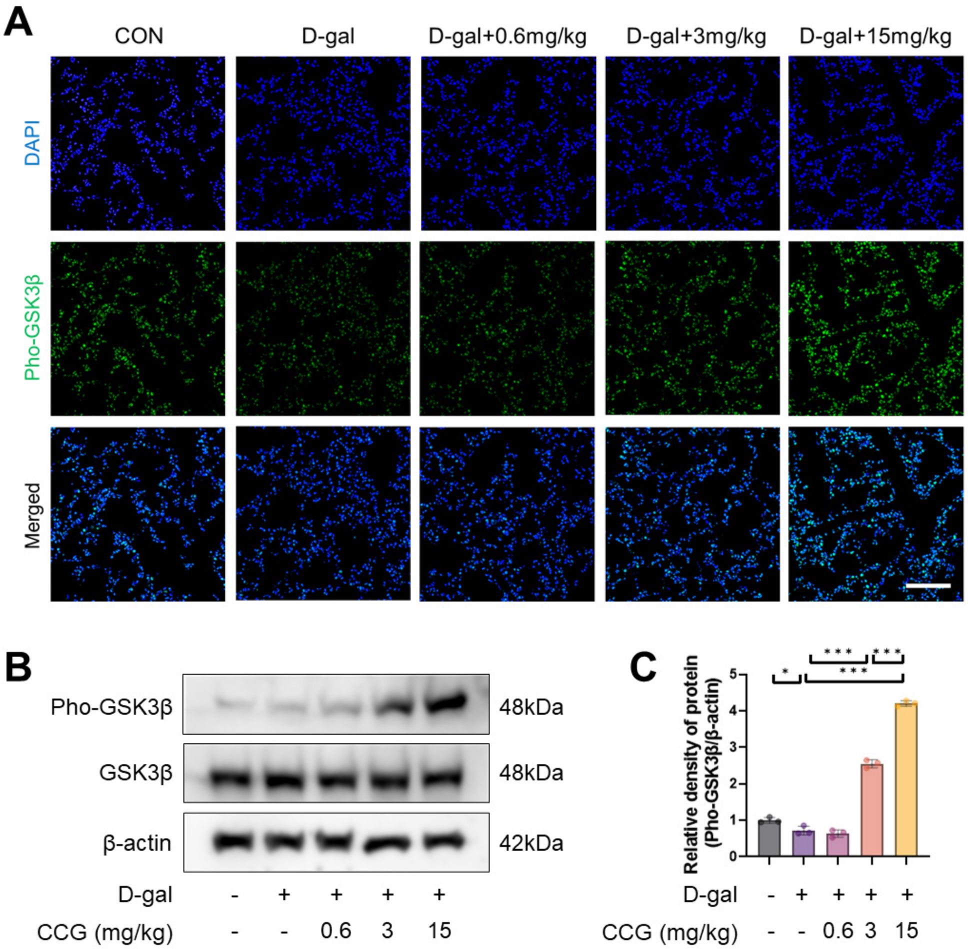

Supplementary Figure S4. CCG promoted the phosphorylation of GSK3β in in the lungs of D-gal-induced mice. (A) Immunofluorescence staining reflecting the expression levels of Pho-GSK3β in the lungs. Green represents Pho-GSK3β, and blue represents DAPI. Scale bars represent 100 μm. (B,C) WB analysis of the protein levels of Pho-GSK3β and GSK3β in vivo. Data represent means ± standard deviation, with each experiment independently repeated at least three times. (* $p < 0.05$, and *** $p < 0.001$).
